# Supplementary figures and images for: Proteomic Characterization of SAS Cell-Derived Extracellular Vesicles in Relation to Both BPA and Neutron Irradiation Doses
Source: Cells. 2023 Jun 6;12(12):1562. doi: 10.3390/cells12121562 (PMC10296566; doi:10.3390/cells12121562)

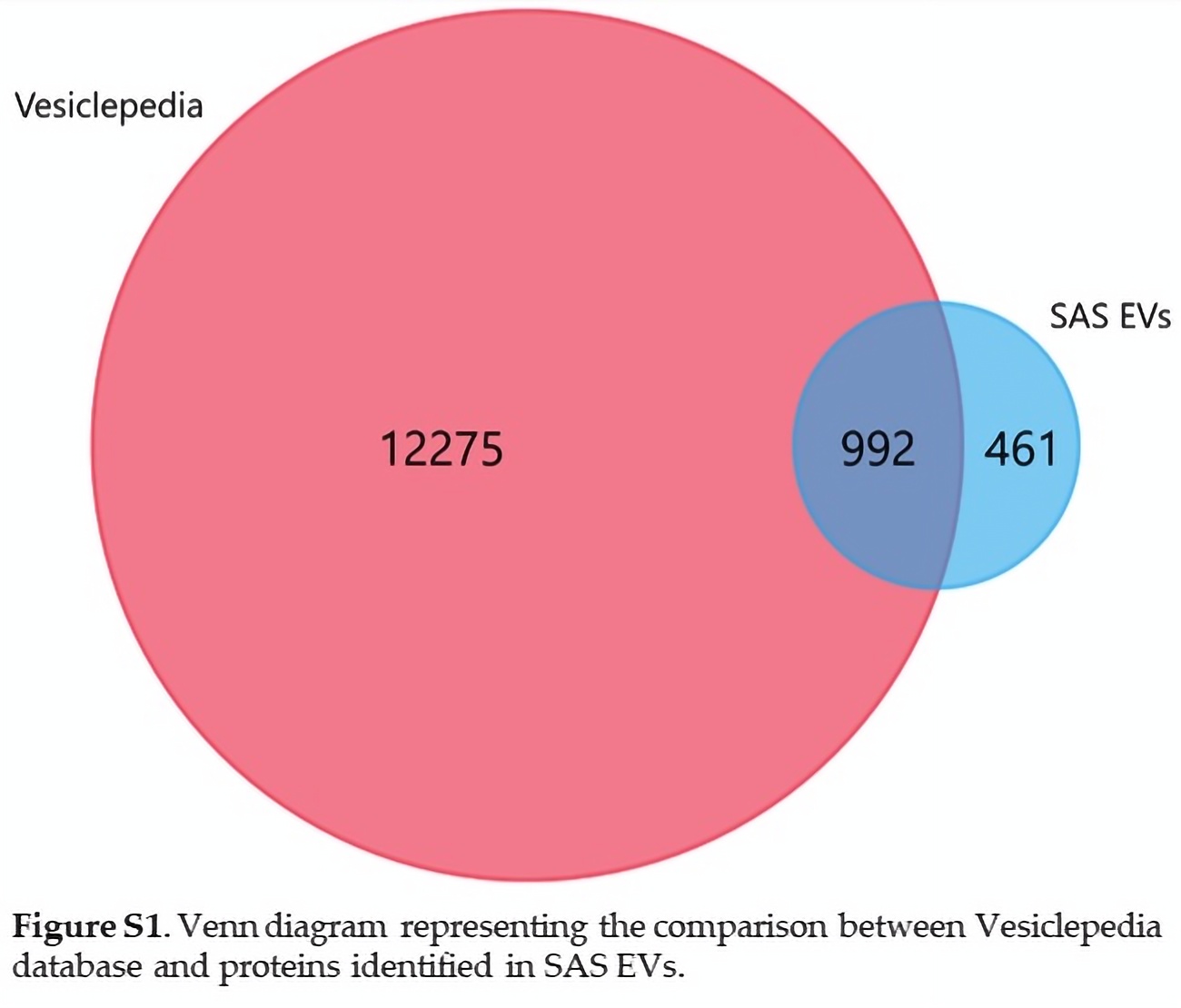

Supplement: Supplementary file 1 [file cells-12-01562-s001.zip › Suppl_Figure_S1.jpg]

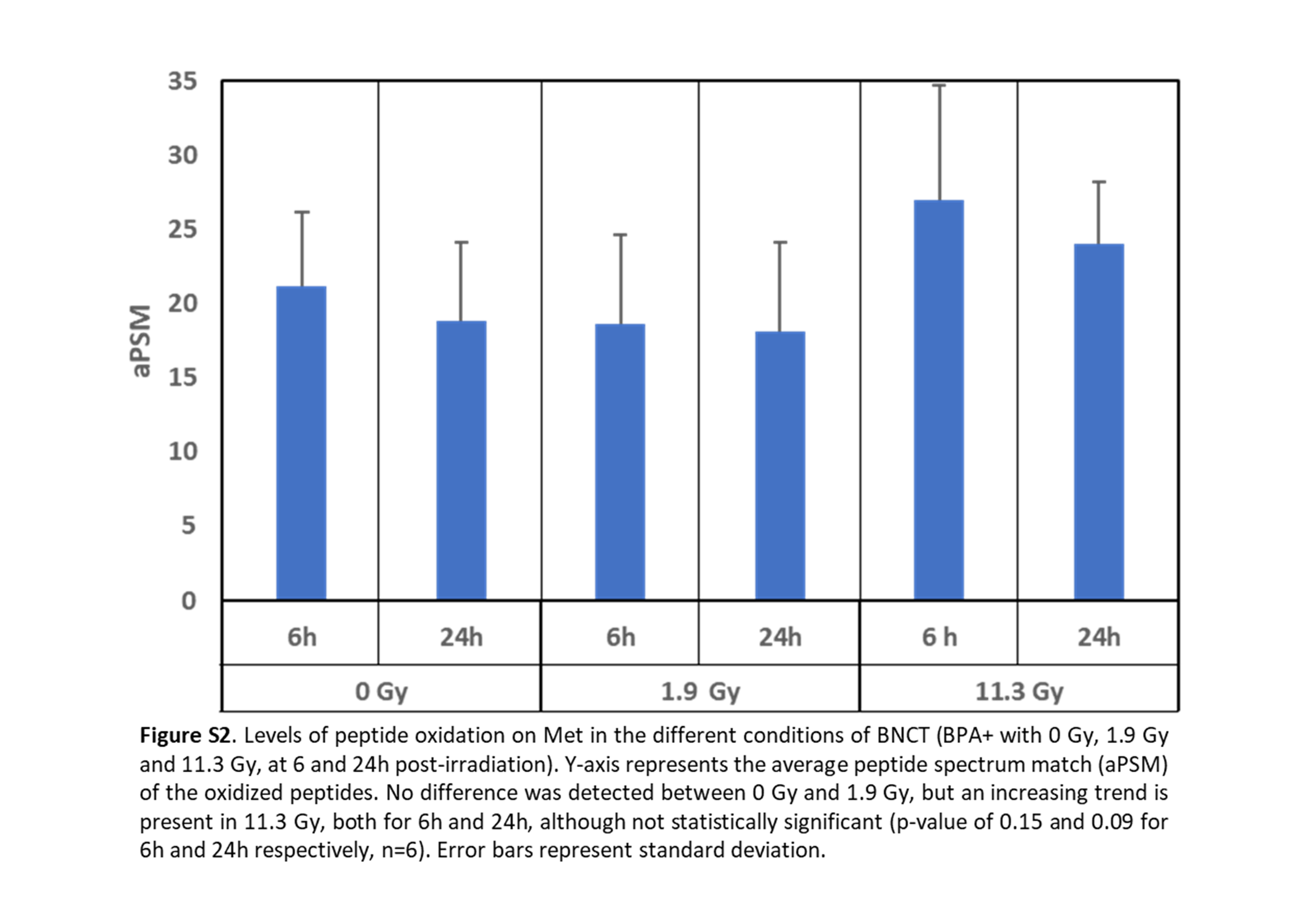

Supplement: Supplementary file 1 [file cells-12-01562-s001.zip › Suppl_Figure_S2.png]
